# Supplementary material for: In Silico Design of miniACE2 Decoys with In Vitro Enhanced Neutralization Activity against SARS-CoV-2, Encompassing Omicron Subvariants
Source: Int J Mol Sci. 2024 Oct 8;25(19):10802. doi: 10.3390/ijms251910802 (PMC11476394; doi:10.3390/ijms251910802)
Supplement: Supplementary file 1 [file ijms-25-10802-s001.zip › Supplementary files/Supplementary figure 5..pdf]

pEcoBP9

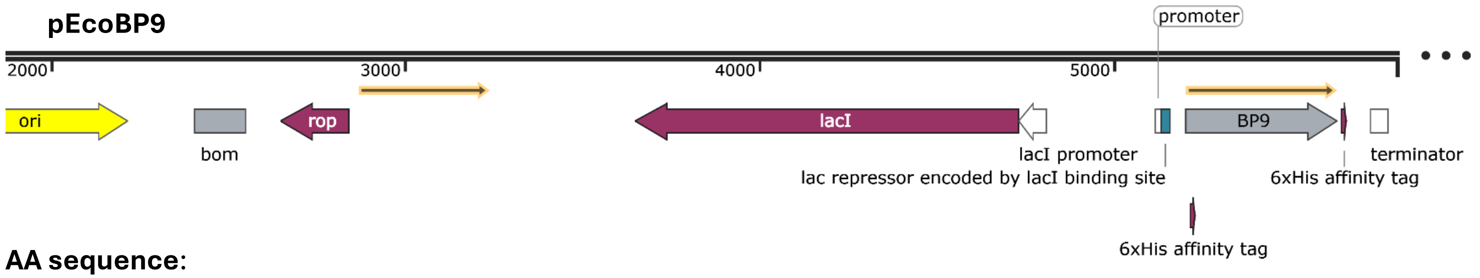

AA sequence:

MRGSHHHHHHTIEEQAKTFLDKFNHEA**ED**LFYQSSLASWNYNTQITEENVQNMNNAGDKWSAFLKEQSTLAQMYPLQEIQQLTVKLQLQALQQNSGSGAVCHPTAWDL
GKGDFRILSCTKVTMDDFLTAHHEMGHIQYDMAY*

Nucleotide sequence:

ATGCGTGGTAGCCATCACCATCATCATCATACCATTGAAGAACAGGCAAAAACCTTCCTGGATAAATTCAATCATGAAGCCGA**G**GA**C**CTGTTTTATCAGAGCAGCCTG
GCAAGCTGGAATTATAACACCCAGATTACCGAAGAAAACGTGCAGAATATGAATAACGCAGGCGATAAATGGTCAGCCTTTCTGAAAGAACAGAGTACCCTGGCACAG
ATGTATCCGCTGCAAGAAATTCAGCAGCTGACCGTTAAACTGCAACTGCAGGCACTGCAGCAGAATAGCGGTAGCGGTGCAGTTTGTCATCCGACCGCATGGGATTTA
GGTAAAGGTGATTTTCGTATTCTGAGCTGCACCAAAGTTACCATGGATGATTTTCTGACCGCACATCATGAAATGGGCCATATTCAGTATGATATGGCCTATTAA

pEcoBP11

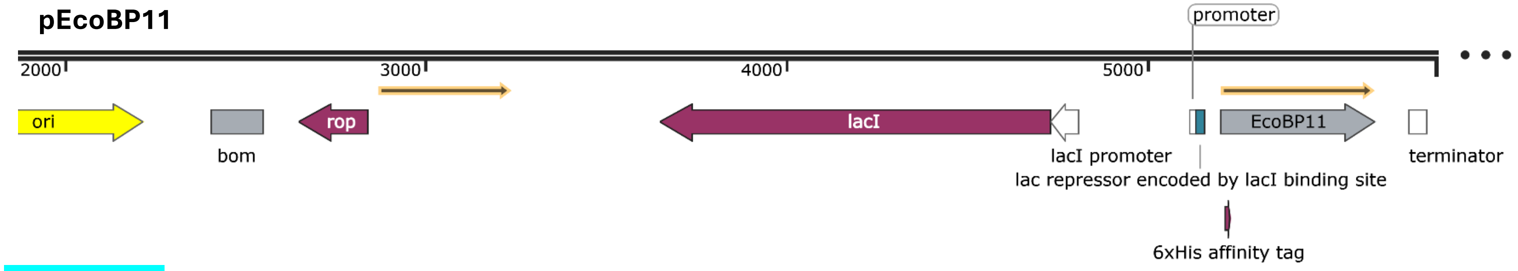

AA sequence:

MRGSHHHHHHTIEEQAKTFLDKFNHEA**DE**LFYQSSLASWNYNTQITEENVQNMNNAGDKWSAFLKEQSTLAQMYPLQEIQQLTVKLQLQALQQNSGSGAVCHPTAWDL
GKGDFRILSCTKVTMDDFLTAHHEMGHIQYDMAY*

Nucleotide sequence:

ATGCGTGGTAGCCATCACCATCATCATCATACCATTGAAGAACAGGCAAAAACCTTCCTGGATAAATTCAATCATGAAGCCGA**C**GA**G**CTGTTTTATCAGAGCAGCCTG
GCAAGCTGGAATTATAACACCCAGATTACCGAAGAAAACGTGCAGAATATGAATAACGCAGGCGATAAATGGTCAGCCTTTCTGAAAGAACAGAGTACCCTGGCACAG

ATGTATCCGCTGCAAGAAATTCAGCAGCTGACCGTTAAACTGCAACTGCAGGCACTGCAGCAGAATAGCGGTAGCGGTGCAGTTTGTCATCCGACCGCATGGGATTTA
GGTAAAGGTGATTTTCGTATTCTGAGCTGCACCAAAGTTACCATGGATGATTTTCTGACCGCACATCATGAAATGGGCCATATTCAGTATGATATGGCCTATTAA

pcDNA-ACE2-His

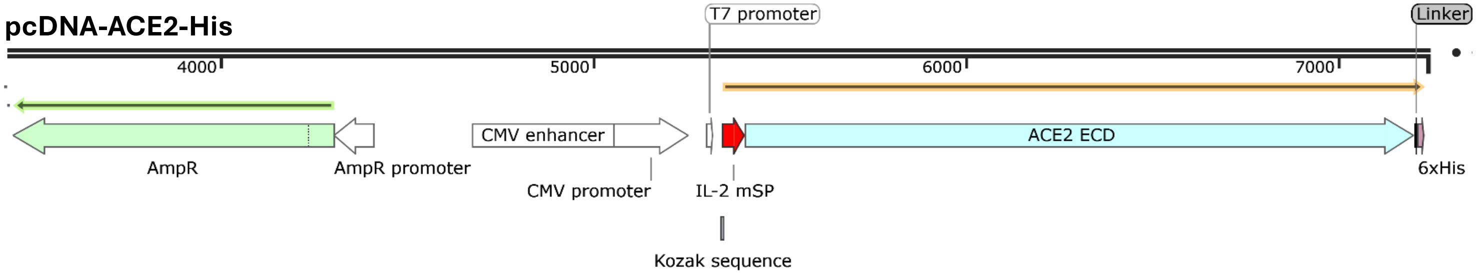

AA sequence:

MARMQLLSCIALSLALVTNSQSTIEEQAKTFLDKFNHEAEDLFYQSSLASWNYNTNITEENVQNMNNAGDKWSAFLKEQSTLAQMYPLQEIQNLTVKLQLQALQQNGS
SVLSEDKSKRLNTILNTMSTIYSTGKVCNPDNPQECLLLEPGLNEIMANSLDYNERLWAWESWRSEVGKQLRPLYEEYVVLKNEMARANHYEDYGDYWRGDYEVNGVD
GYDYSRGQLIEDVEHTFEEIKPLYEHLHAYVRAKLMNAYPSYISPIGCLPAHLLGDMWGRFWTNLYSLTVPFGQKPNIDVTDAMVDQAWDAQRIFKEAEKFFVSVGLP
NMTQGFWENSMLTDPGNVQKAVCHPTAWDLGKGDFRILMCTKVTMDDFLTAHHEMGHIQYDMAYAAQPFLLRNGANEGFHEAVGEIMSLSAATPKHLKSIGLLSPDFQ
EDNETEINFLLKQALTIVGTLPFTYMLEKWRWMVFKGEIPKDQWMKKWWEMKREIVGVVEPVPHDETYCDPASLFHVSNDYSFIRYYTRTLYQFQFQEALCQAAKHEG
PLHKCDISNSTEAGQKLFNMLRLGKSEPWTLALENVVGAKNMNVRPLLNYFEPLFTWLKDQNKNSFVGWSTDWSPYADGSGHHHHHH*

Nucleotide sequence:

ATGGCTAGGATGCAACTCCTGTCTTGCATTGCACTAAGTCTTGCACTTGTCACGAATTCGCAGTCCACCATTGAGGAACAGGCCAAGACATTTTTGGACAAGTTTAAC
CACGAAGCCGAAGACCTGTTCTATCAAAGTTCACTTGCTTCTTGGAATTATAACACCAATATTACTGAAGAGAATGTCCAAAACATGAATAATGCTGGGGACAAATGG
TCTGCCTTTTTAAAGGAACAGTCCACACTTGCCCAAATGTATCCACTACAAGAAATTCAGAATCTCACAGTCAAGCTTCAGCTGCAGGCTCTTCAGCAAAATGGGTCT
TCAGTGCTCTCAGAAGACAAGAGCAAACGGTTGAACACAATTCTAAATACAATGAGCACCATCTACAGTACTGGAAAAGTTTGTAACCCAGATAATCCACAAGAATGC
TTATTACTTGAACCAGGTTTGAATGAAATAATGGCAAACAGTTTAGACTACAATGAGAGGCTCTGGGCTTGGGAAAGCTGGAGATCTGAGGTCGGCAAGCAGCTGAGG
CCATTATATGAAGAGTATGTGGTCTTGAAAAATGAGATGGCAAGAGCAAATCATTATGAGGACTATGGGGATTATTGGAGAGGAGACTATGAAGTAAATGGGGTAGAT
GGCTATGACTACAGCCGCGGCCAGTTGATTGAAGATGTGGAACATACCTTTGAAGAGATTAAACCATTATATGAACATCTTCATGCCTATGTGAGGGCAAAGTTGATG
AATGCCTATCCTTCCTATATCAGTCCAATTGGATGCCTCCCTGCTCATTTGCTTGGTGATATGTGGGGTAGATTTTGGACAAATCTGTACTCTTTGACAGTTCCCTTT
GGACAGAAACCAAACATAGATGTTACTGATGCAATGGTGGACCAGGCCTGGGATGCACAGAGAATATTCAAGGAGGCCGAGAAGTTCTTTGTATCTGTTGGTCTTCCT
AATATGACTCAAGGATTCTGGGAAAATTCCATGCTAACGGACCCAGGAAATGTTCAGAAAGCAGTCTGCCATCCCACAGCTTGGGACCTGGGGAAGGGCGACTTCAGG
ATTCTTATGTGCACAAAGGTGACAATGGACGACTTCCTGACAGCTCATCATGAGATGGGGCATATCCAGTATGATATGGCATATGCTGCACAACCTTTTCTGCTAAGA
AATGGAGCTAATGAAGGATTCCATGAAGCTGTTGGGGAAATCATGTCACTTTCTGCAGCCACACCTAAGCATTTAAAATCCATTGGTCTTCTGTCACCCGATTTTCAA
GAAGACAATGAAACAGAAATAAACTTCCTGCTCAAACAAGCACTCACGATTGTTGGGACTCTGCCATTTACTTACATGTTAGAGAAGTGGAGGTGGATGGTCTTTAAA

GGGGAAATTCCCAAAGACCAGTGGATGAAAAAGTGGTGGGAGATGAAGCGAGAGATAGTTGGGGTGGTGGAACCTGTGCCCCATGATGAAACATACTGTGACCCCGCA
TCTCTGTTCCATGTTTCTAATGATTACTCATTCATTCGATATTACACAAGGACCCTTTACCAATTCCAGTTTCAAGAAGCACTTTGTCAAGCAGCTAAACATGAAGGC
CCTCTGCACAAATGTGACATCTCAAACTCTACAGAAGCTGGACAGAAACTGTTCAATATGCTGAGGCTTGGAAAATCAGAACCCTGGACCCTAGCATTGGAAAATGTT
GTAGGAGCAAAGAACATGAATGTAAGGCCACTGCTCAACTACTTTGAGCCCTTATTTACCTGGCTGAAAGACCAGAACAAGAATTCTTTTGTGGGATGGAGTACCGAC
TGGAGTCCATATGCAGACGGGTCCGGTCACCACCATCACCATCATTGA
