# Supplementary material for: In Silico Design of miniACE2 Decoys with In Vitro Enhanced Neutralization Activity against SARS-CoV-2, Encompassing Omicron Subvariants
Source: Int J Mol Sci. 2024 Oct 8;25(19):10802. doi: 10.3390/ijms251910802 (PMC11476394; doi:10.3390/ijms251910802)
Supplement: Supplementary file 1 [file ijms-25-10802-s001.zip › Supplementary files/Supplementary files ijms-3233904.docx]

Article

*In-silico* design of miniACE2 decoys with *In-vitro* enhanced neutralization activity against SARS-CoV-2, *encompassing Omicron Subvariants.*

Jenny Andrea Arévalo-Romero ^1,2¶^, Gina López-Cantillo ^1¶^, Sara Moreno-Jiménez ^1^, Íñigo Marcos-Alcalde ^3^, David Ros-Pardo ^3^, Bernardo Armando Camacho ^1^, Paulino Gómez-Puertas ^3,^* and Cesar A. Ramirez-Segura ^1,^*

| **Citation:** To be added by editorial staff during production.  Academic Editor: Firstname Lastname  Received: date  Revised: date  Accepted: date  Published: date  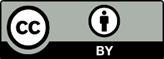  **Copyright:** © 2024 by the authors. Submitted for possible open access publication under the terms and conditions of the Creative Commons Attribution (CC BY) license (https://creativecommons.org/licenses/by/4.0/). |
| --- |

^1^ Unidad de Ingeniería Celular y Molecular, Instituto Distrital de Ciencia, Biotecnología e Innovación en Salud, IDCBIS, 111611, Bogotá DC, Colombia.

^2^ Instituto de Errores Innatos del Metabolismo, Facultad de Ciencias, Pontificia Universidad Javeriana, 110231, Bogotá, D.C., Colombia.

^3^ Grupo de Modelado Molecular del Centro de Biología Molecular Severo Ochoa, 14 CSIC-UAM, 28049 Madrid, Spain.

***** Correspondence: email: CARS cramirez@idcbis.org.co; cramirezbiologo@gmail.com: PGP pagomez@cbm.csic.es

^¶^ These authors contributed equally to this work

**Supplementary Materials:** The following supporting information can be downloaded at: [www.mdpi.com/Figure/s1](http://www.mdpi.com/Figure/s1), Figure S1: Representation of the most promising miniACE2 candidates selected for Molecular Dynamics (MD) simulation; [www.mdpi.com/Figure/s2](http://www.mdpi.com/Figure/s2), Figure S2: Stability of BPs structure in interaction with the RBD; [www.mdpi.com/Figure/s3](http://www.mdpi.com/Figure/s3), Figure S3: Multiple sequence alignment of ACE2, BP2, BP9 and BP11; [www.mdpi.com/Figure/s4](http://www.mdpi.com/Figure/s4), Figure S4: Method for Extracting ACE2 Regions Interacting with the RBD; [www.mdpi.com/Figure/s5](http://www.mdpi.com/Figure/s5), Figure S5: Plasmids construction, Nucleotide and Amino Acid Sequence; [www.mdpi.com/Figure/s6](http://www.mdpi.com/Figure/s6), Figure S6: BP9 and BP11 proteins expressed in E. coli analyzed by SDS-PAGE and western blotting; [www.mdpi.com/Figure/s7](http://www.mdpi.com/Figure/s7), Figure S7: Western blot analysis confirming the expression of hACE2 protein; [www.mdpi.com/Table/s1](http://www.mdpi.com/Table/s1), Table S1: Values of the root mean square deviation (RMSD) of Blocking protein (BP) structures in interaction with the Receptor Binding Domain (RBD); [www.mdpi.com/Table/s2](http://www.mdpi.com/Table/s2), Table S2: Human serum samples without neutralizing antibodies against SARS-CoV-2; [www.mdpi.com/Table/s3](http://www.mdpi.com/Table/s3), Table S3: Promising Blocking Proteins (BPs) selected for further study.

**
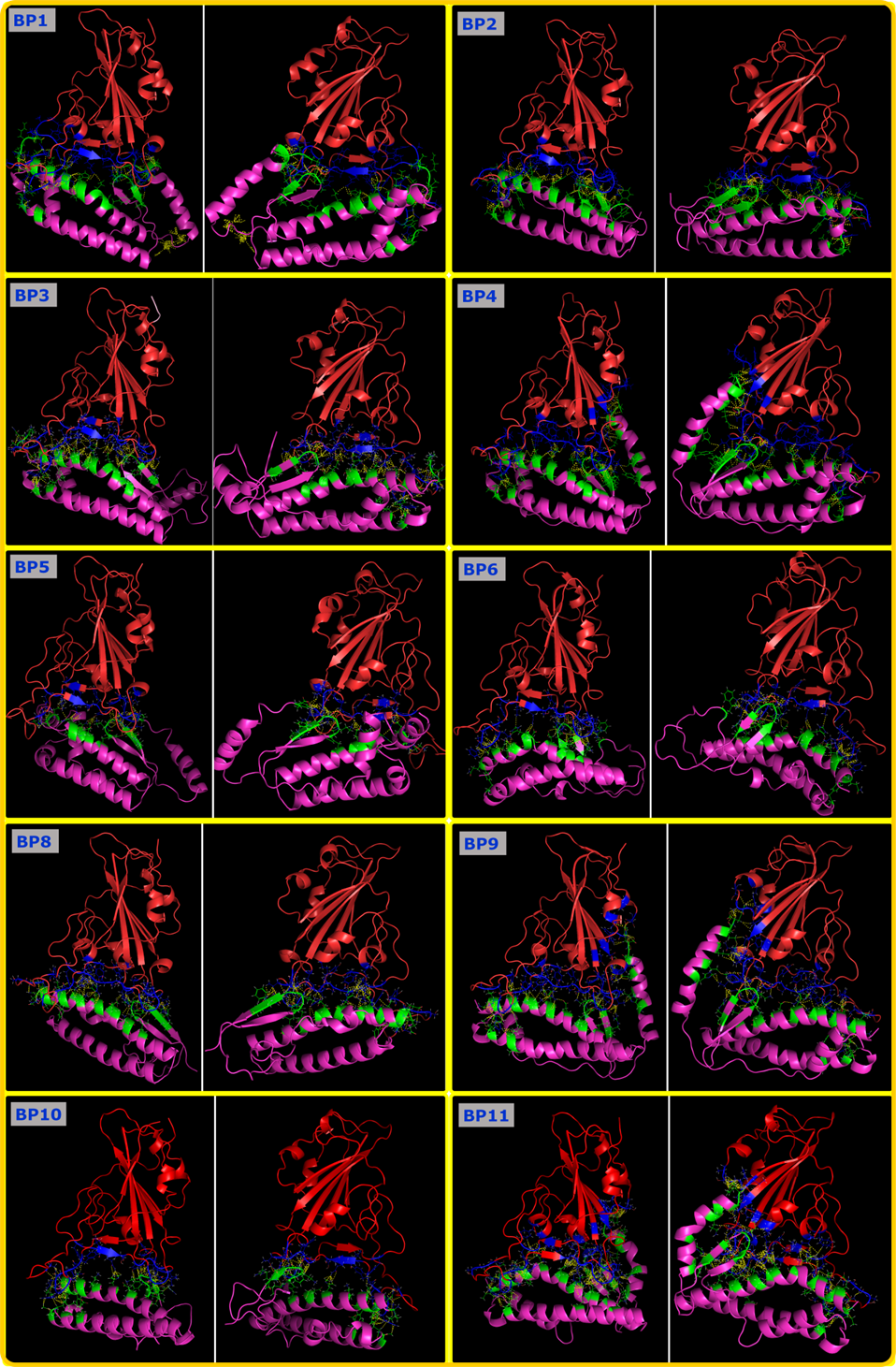
**

**Figure S1.** Representation of the most promising miniACE2 candidates selected for Molecular Dynamics (MD) simulation. Each structure is depicted from two different angles to provide a comprehensive view of their interaction with the Receptor Binding Domain (RBD). The images show the Binding Partners (BPs) in fuchsia and the RBD in red. The relevant regions involved in the binding interactions are highlighted: dark blue on the RBD and green on the BPs.


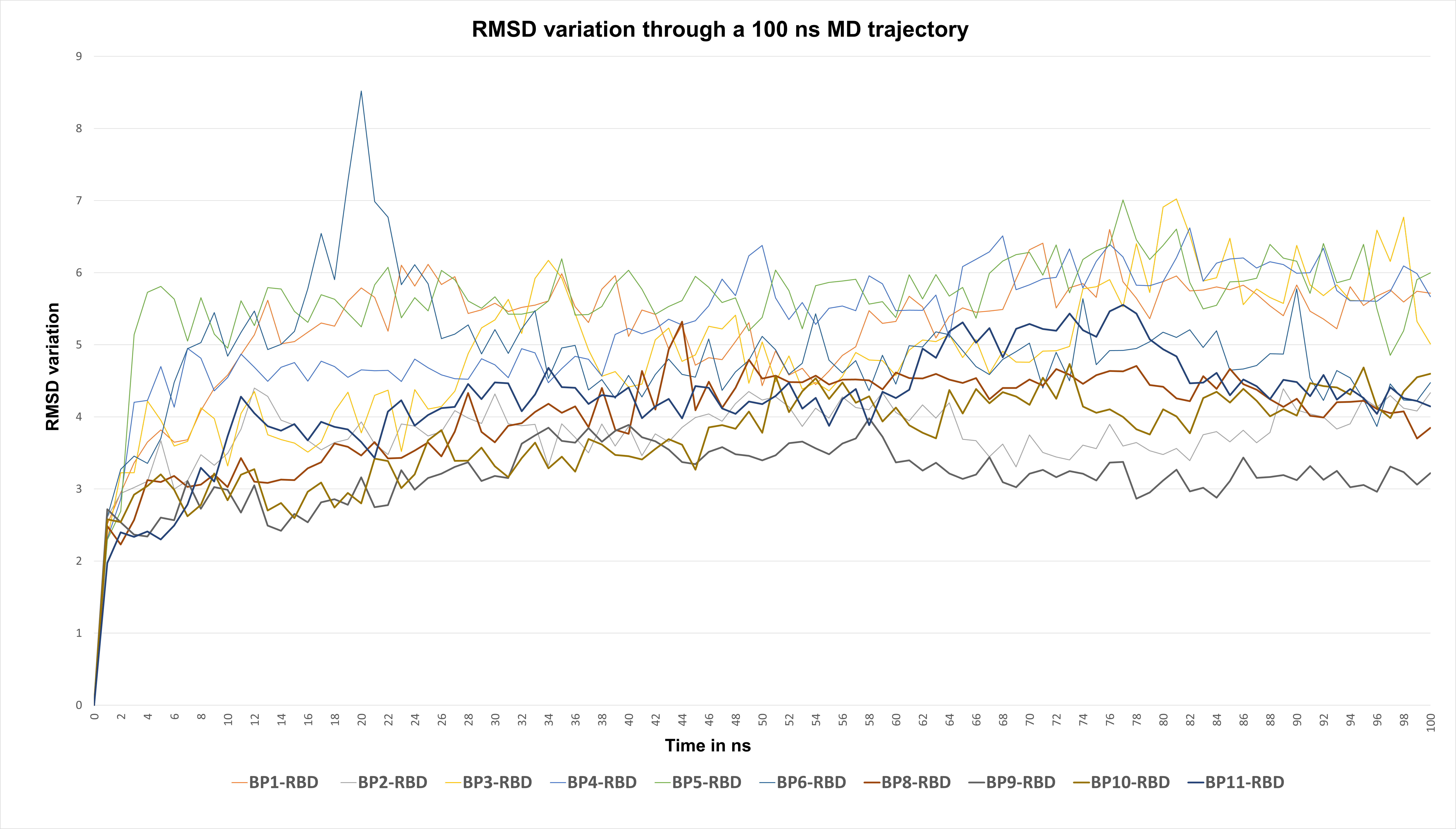


**Figure S2.** Stability of BPs structure in interaction with the RBD, was measured by monitoring the RMSD variation through 100 ns MD trajectory. Data comes from Supplementary table 1**.**


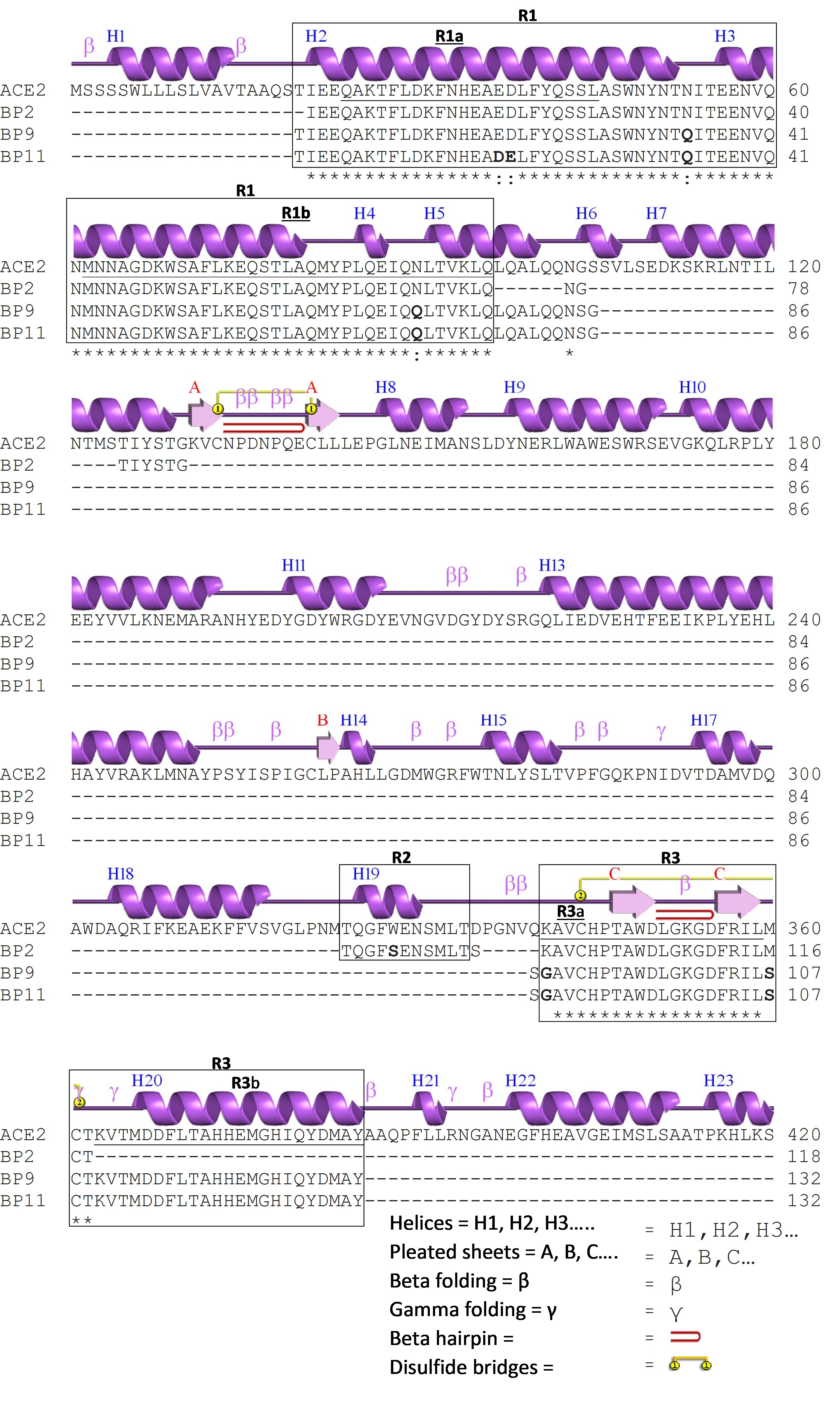


**Figure S3.** Multiple sequence alignment of ACE2, BP2, BP9 and BP11. R1, R2 and R3 are segments of ACE2 sequence included in the design of the miniACE2.

**
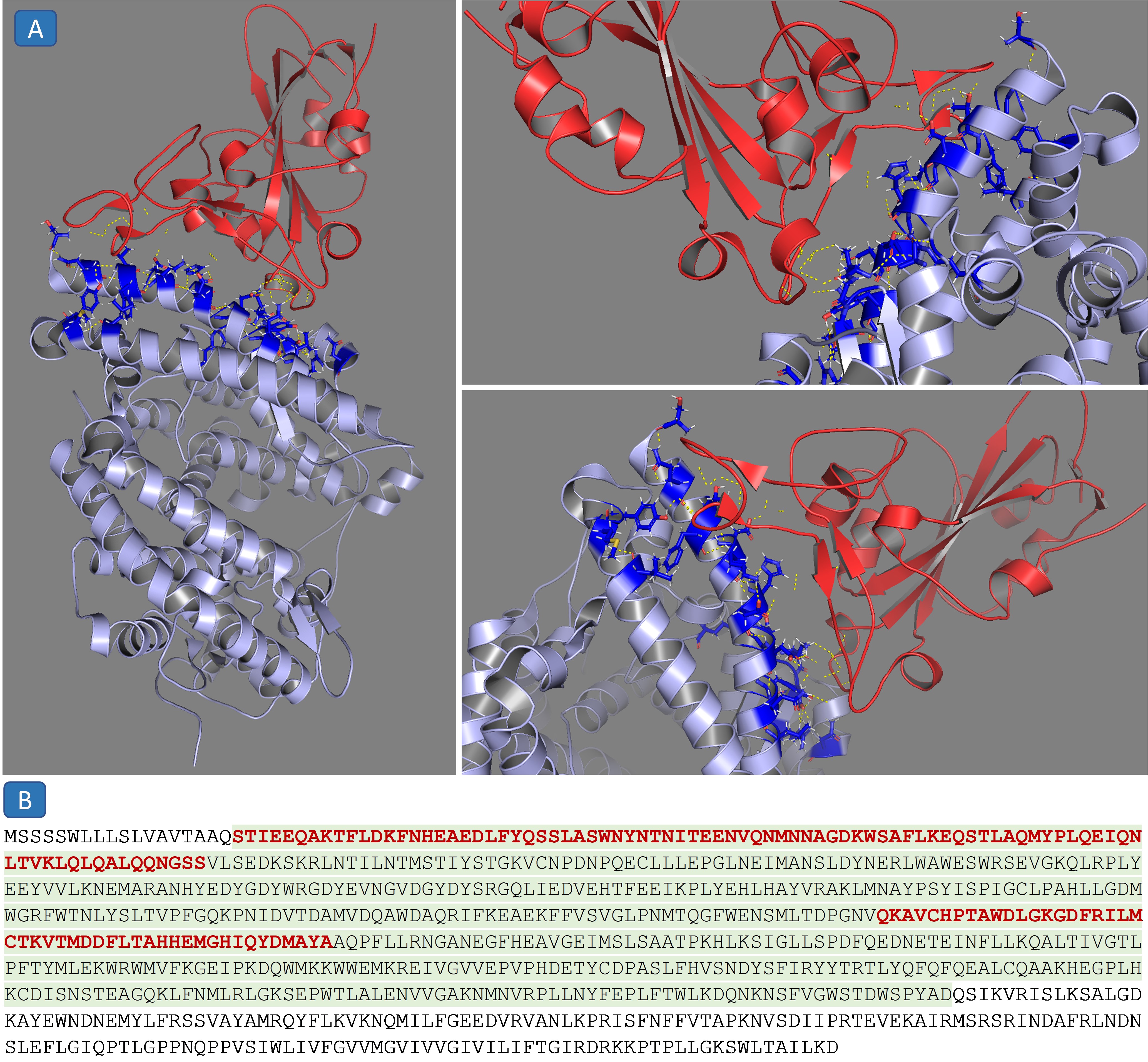
**

**Figure S4.** Method for Extracting ACE2 Regions Interacting with the RBD. A – Crystal structure of the SARS-CoV-2 spike receptor-binding domain (RBD) complexed with ACE2 (PDB: 6M0J) was analyzed using the PyMOL graphical environment [29]. Three images in panel A depict different angles, highlighting the interactions between ACE2 and RBD (indicated by yellow dots). B – The ACE2 amino acid sequence (from the 6M0J_A structure) is highlighted in green, with the relevant regions involved in binding to RBD highlighted in red sequence.


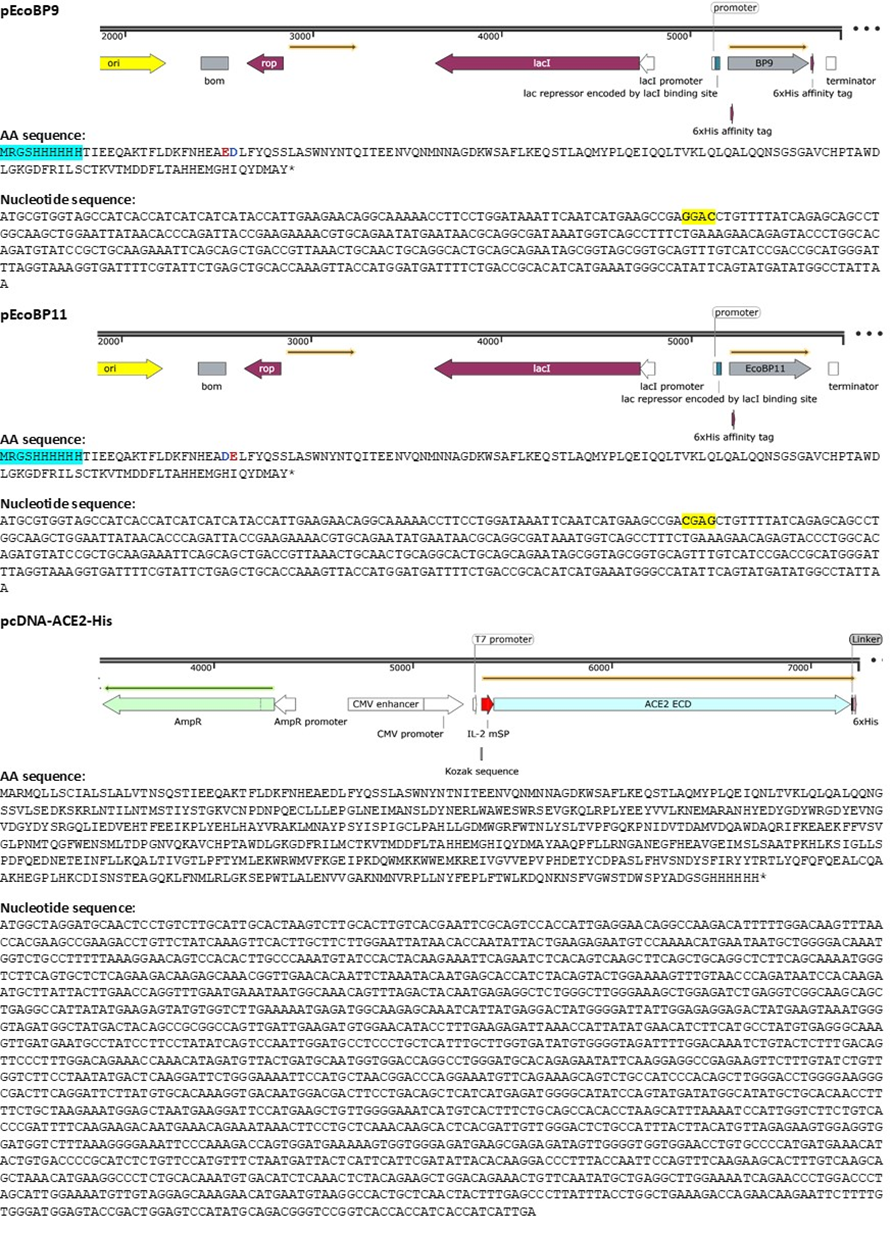


**Figure S5.** Plasmids construction, Nucleotide and Amino Acid Sequence.


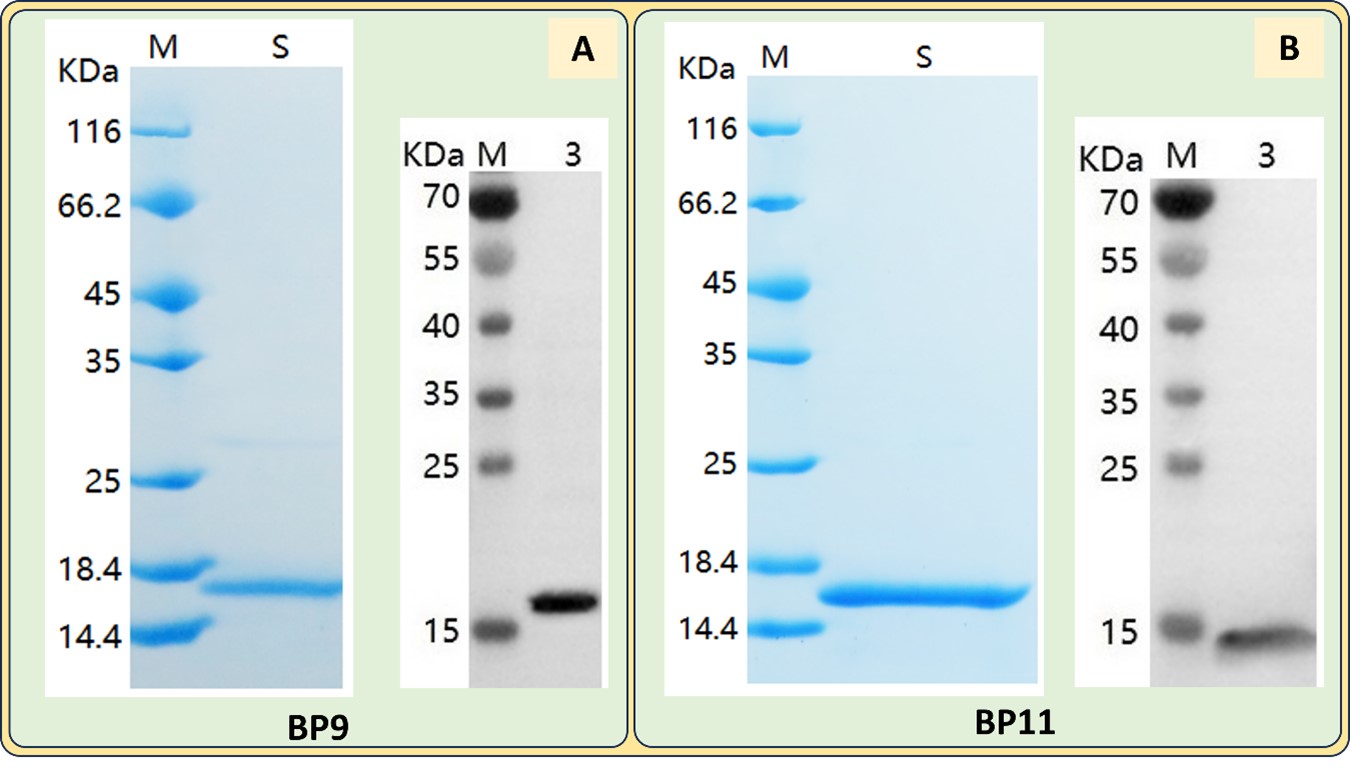


**Figure S6.** BP9 and BP11 proteins expressed in E. coli analyzed by SDS-PAGE and western blotting. SDS-PAGE (A) and western blotting confirmation (B).


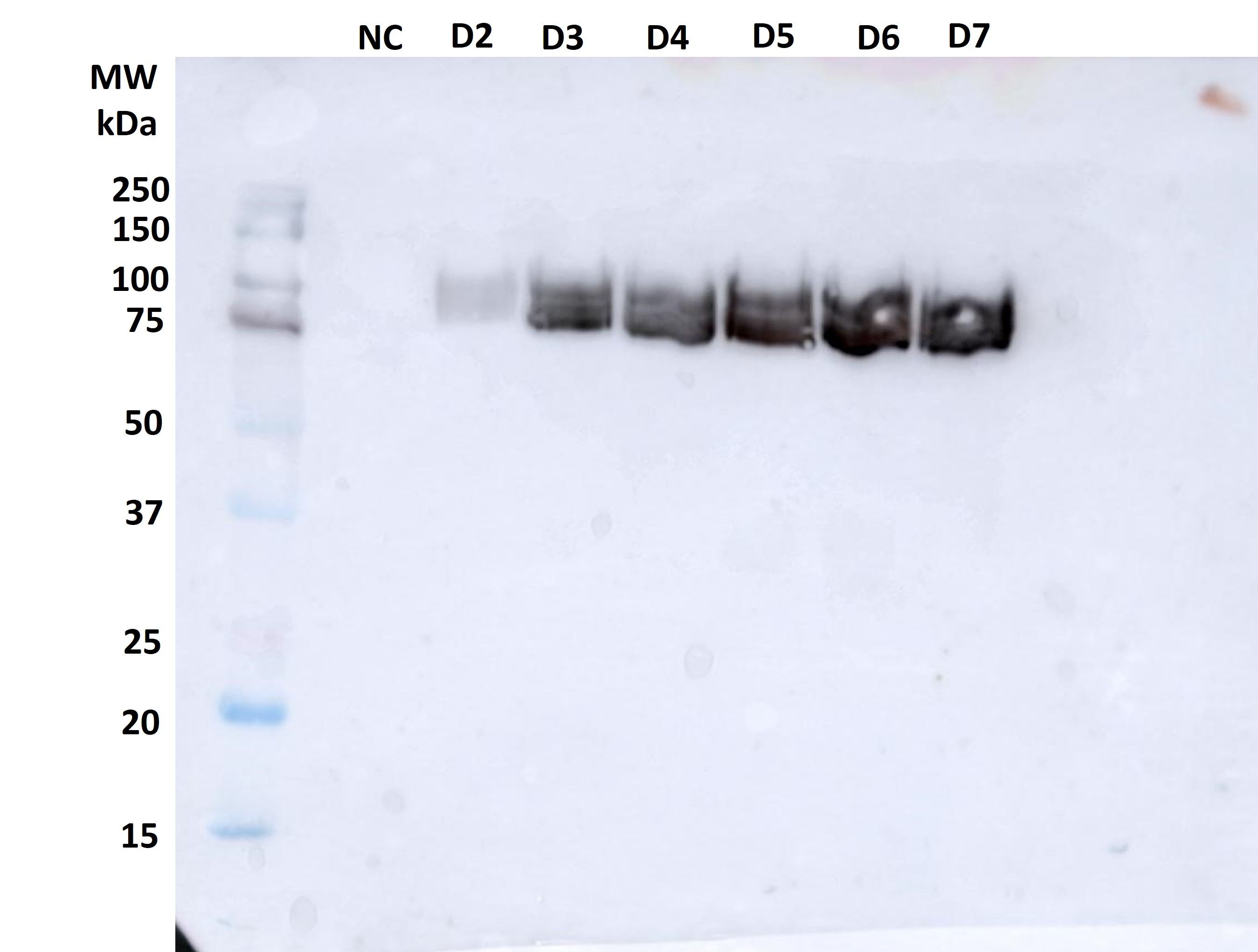


**Figure S7.** Western blot analysis confirming the expression of hACE2 protein in the supernatant of Expi293F™ cell line cultures.

**Table S1.** Values of the root mean square deviation (RMSD) of Blocking protein (BP) structures in interaction with the Receptor Binding Domain (RBD) through a 100 ns Molecular Dynamic (MD) trajectory.

| **RMSD variation in BP structure ►**  **Time in ns ▼** | **BP1-RBD** | **BP2-RBD** | **BP3-RBD** | **BP4-RBD** | **BP5-RBD** | **BP6-RBD** | **BP8-RBD** | **BP9-RBD** | **BP10-RBD** | **BP11-RBD** |
| --- | --- | --- | --- | --- | --- | --- | --- | --- | --- | --- |
| 0 | 0 | 0 | 0 | 0 | 0 | 0 | 0 | 0 | 0 | 0 |
| 1 | 2.5140 | 2.6112 | 2.3051 | 2.3128 | 2.2952 | 2.6146 | 2.4854 | 2.7183 | 2.5781 | 1.9745 |
| 2 | 2.9485 | 2.9376 | 3.2254 | 2.8597 | 2.6906 | 3.2725 | 2.2302 | 2.5386 | 2.5402 | 2.3991 |
| 3 | 3.3620 | 3.0202 | 3.2249 | 4.2012 | 5.1416 | 3.4571 | 2.5699 | 2.3669 | 2.9196 | 2.3361 |
| 4 | 3.6500 | 3.1072 | 4.2180 | 4.2293 | 5.7287 | 3.3547 | 3.1218 | 2.3396 | 3.0402 | 2.4096 |
| 5 | 3.8196 | 3.6704 | 3.9509 | 4.6977 | 5.8108 | 3.7018 | 3.0953 | 2.6016 | 3.2018 | 2.3006 |
| 6 | 3.6489 | 2.9997 | 3.5967 | 4.1345 | 5.6337 | 4.4799 | 3.1807 | 2.5666 | 2.9907 | 2.4913 |
| 7 | 3.6811 | 3.1156 | 3.6615 | 4.9504 | 5.0528 | 4.9468 | 3.0297 | 3.1099 | 2.6230 | 2.7829 |
| 8 | 4.0892 | 3.4745 | 4.1248 | 4.8145 | 5.6538 | 5.0300 | 3.0594 | 2.7254 | 2.7794 | 3.2930 |
| 9 | 4.3982 | 3.3281 | 3.9765 | 4.3619 | 5.1483 | 5.4453 | 3.2019 | 3.0263 | 3.2137 | 3.1034 |
| 10 | 4.5800 | 3.4946 | 3.3178 | 4.5453 | 4.9541 | 4.8422 | 3.0258 | 2.9887 | 2.8445 | 3.7292 |
| 11 | 4.8646 | 3.8297 | 4.0478 | 4.8723 | 5.6108 | 5.1720 | 3.4279 | 2.6733 | 3.1972 | 4.2794 |
| 12 | 5.1394 | 4.3992 | 4.3518 | 4.6874 | 5.2657 | 5.4670 | 3.1021 | 3.0506 | 3.2734 | 4.0520 |
| 13 | 5.6181 | 4.2840 | 3.7525 | 4.4934 | 5.7925 | 4.9335 | 3.0835 | 2.4923 | 2.7007 | 3.8702 |
| 14 | 5.0133 | 3.9552 | 3.6846 | 4.6889 | 5.7742 | 5.0047 | 3.1301 | 2.4202 | 2.8046 | 3.8095 |
| 15 | 5.0451 | 3.8850 | 3.6363 | 4.7538 | 5.4627 | 5.1860 | 3.1225 | 2.6542 | 2.5953 | 3.8993 |
| 16 | 5.1744 | 3.6736 | 3.5120 | 4.4960 | 5.3111 | 5.7786 | 3.2868 | 2.5388 | 2.9604 | 3.6734 |
| 17 | 5.3018 | 3.5418 | 3.6510 | 4.7729 | 5.6953 | 6.5437 | 3.3698 | 2.8153 | 3.0886 | 3.9309 |
| 18 | 5.2577 | 3.6429 | 4.0780 | 4.6975 | 5.6327 | 5.9039 | 3.6308 | 2.8588 | 2.7422 | 3.8621 |
| 19 | 5.6023 | 3.6885 | 4.3414 | 4.5486 | 5.4381 | 7.2726 | 3.5848 | 2.7817 | 2.9436 | 3.8243 |
| 20 | 5.7881 | 3.9291 | 3.7794 | 4.6527 | 5.2485 | 8.5205 | 3.4646 | 3.1621 | 2.8003 | 3.6517 |
| 21 | 5.6571 | 3.6256 | 4.2988 | 4.6398 | 5.8309 | 6.9856 | 3.6500 | 2.7474 | 3.4210 | 3.4311 |
| 22 | 5.1896 | 3.4787 | 4.3715 | 4.6447 | 6.0757 | 6.7696 | 3.4236 | 2.7749 | 3.3873 | 4.0729 |
| 23 | 6.1031 | 3.9023 | 3.5214 | 4.4900 | 5.3744 | 5.8348 | 3.4303 | 3.2604 | 3.0127 | 4.2308 |
| 24 | 5.8154 | 3.8723 | 4.3780 | 4.8023 | 5.6524 | 6.1094 | 3.5315 | 2.9910 | 3.2027 | 3.8788 |
| 25 | 6.1164 | 3.7352 | 4.1087 | 4.6816 | 5.4707 | 5.8459 | 3.6446 | 3.1517 | 3.6748 | 4.0261 |
| 26 | 5.8366 | 3.7964 | 4.1433 | 4.5831 | 6.0300 | 5.0844 | 3.4523 | 3.2107 | 3.8156 | 4.1224 |
| 27 | 5.9445 | 4.0870 | 4.3554 | 4.5282 | 5.9005 | 5.1485 | 3.7936 | 3.3058 | 3.3893 | 4.1393 |
| 28 | 5.4342 | 3.9819 | 4.8786 | 4.5217 | 5.6068 | 5.2763 | 4.3316 | 3.3698 | 3.3933 | 4.4565 |
| 29 | 5.4847 | 3.9088 | 5.2343 | 4.8053 | 5.5064 | 4.8728 | 3.7921 | 3.1101 | 3.5720 | 4.2470 |
| 30 | 5.5761 | 4.3166 | 5.3450 | 4.7229 | 5.6657 | 5.2112 | 3.6481 | 3.1805 | 3.3155 | 4.4778 |
| 31 | 5.4606 | 3.9038 | 5.6281 | 4.5446 | 5.4240 | 4.8801 | 3.8751 | 3.1522 | 3.1626 | 4.4645 |
| 32 | 5.5177 | 3.8774 | 5.1579 | 4.9469 | 5.4250 | 5.2210 | 3.9112 | 3.6279 | 3.4275 | 4.0783 |
| 33 | 5.5493 | 3.8965 | 5.9205 | 4.8877 | 5.4644 | 5.4726 | 4.0674 | 3.7441 | 3.6421 | 4.3122 |
| 34 | 5.6085 | 3.3182 | 6.1712 | 4.4731 | 5.6084 | 4.5321 | 4.1814 | 3.8491 | 3.2870 | 4.6788 |
| 35 | 5.9858 | 3.9039 | 5.9283 | 4.6680 | 6.1925 | 4.9549 | 4.0571 | 3.6657 | 3.4464 | 4.4120 |
| 36 | 5.5275 | 3.7162 | 5.4345 | 4.8410 | 5.4129 | 4.9925 | 4.1447 | 3.6413 | 3.2404 | 4.4029 |
| 37 | 5.3088 | 3.5009 | 4.9321 | 4.7995 | 5.4202 | 4.3729 | 3.8563 | 3.8451 | 3.6942 | 4.1782 |
| 38 | 5.7703 | 3.9020 | 4.5642 | 4.5684 | 5.5310 | 4.5164 | 4.4009 | 3.6545 | 3.6150 | 4.3027 |
| 39 | 5.9581 | 3.5960 | 4.6274 | 5.1430 | 5.8518 | 4.2564 | 3.8205 | 3.8068 | 3.4718 | 4.2773 |
| 40 | 5.1155 | 3.8662 | 4.4130 | 5.2273 | 6.0332 | 4.5745 | 3.7646 | 3.8882 | 3.4557 | 4.4090 |
| 41 | 5.4822 | 3.4638 | 4.4556 | 5.1530 | 5.7711 | 4.2773 | 4.6364 | 3.7161 | 3.4080 | 3.9823 |
| 42 | 5.4213 | 3.7638 | 5.0680 | 5.2169 | 5.4216 | 4.5870 | 4.1028 | 3.6607 | 3.5561 | 4.1447 |
| 43 | 4.9447 | 3.6544 | 5.2329 | 5.3537 | 5.5309 | 4.8010 | 4.9352 | 3.5471 | 3.6895 | 4.2480 |
| 44 | 5.2681 | 3.8534 | 4.7712 | 5.2745 | 5.6133 | 4.5888 | 5.3207 | 3.3729 | 3.6140 | 3.9795 |
| 45 | 4.7167 | 3.9913 | 4.8593 | 5.3325 | 5.9490 | 4.5522 | 4.0922 | 3.3446 | 3.2664 | 4.4266 |
| 46 | 4.8197 | 4.0401 | 5.2565 | 5.5404 | 5.7980 | 5.0816 | 4.4865 | 3.5142 | 3.8545 | 4.4037 |
| 47 | 4.7946 | 3.9390 | 5.2191 | 5.9114 | 5.5857 | 4.3679 | 4.1257 | 3.5800 | 3.8856 | 4.1172 |
| 48 | 5.0436 | 4.1826 | 5.4114 | 5.6826 | 5.6504 | 4.6232 | 4.4028 | 3.4808 | 3.8347 | 4.0410 |
| 49 | 5.3067 | 4.3522 | 4.4679 | 6.2343 | 5.1929 | 4.7846 | 4.7919 | 3.4604 | 4.0741 | 4.2132 |
| 50 | 4.4341 | 4.2340 | 5.0401 | 6.3769 | 5.3783 | 5.1172 | 4.5314 | 3.3970 | 3.7788 | 4.1769 |
| 51 | 4.9153 | 4.2799 | 4.4851 | 5.6503 | 6.0350 | 4.9304 | 4.5719 | 3.4656 | 4.5641 | 4.2894 |
| 52 | 4.5789 | 4.1388 | 4.8455 | 5.3490 | 5.7511 | 4.5901 | 4.4815 | 3.6349 | 4.0661 | 4.4722 |
| 53 | 4.6735 | 3.8677 | 4.3858 | 5.5852 | 5.2230 | 4.7434 | 4.4798 | 3.6579 | 4.3546 | 4.1148 |
| 54 | 4.4481 | 4.1206 | 4.4783 | 5.2837 | 5.8191 | 5.4287 | 4.5718 | 3.5621 | 4.5360 | 4.2640 |
| 55 | 4.6362 | 3.9821 | 4.3616 | 5.5062 | 5.8653 | 4.7873 | 4.4482 | 3.4819 | 4.2502 | 3.8754 |
| 56 | 4.8529 | 4.2764 | 4.5632 | 5.5373 | 5.8852 | 4.6105 | 4.5165 | 3.6310 | 4.4779 | 4.2470 |
| 57 | 4.9715 | 4.1296 | 4.8938 | 5.4729 | 5.9081 | 4.7803 | 4.5177 | 3.7003 | 4.1959 | 4.3851 |
| 58 | 5.4722 | 4.0982 | 4.7900 | 5.9558 | 5.5637 | 4.3630 | 4.5076 | 3.9803 | 4.2855 | 3.8853 |
| 59 | 5.2980 | 4.3377 | 4.7806 | 5.8468 | 5.5980 | 4.8540 | 4.3823 | 3.7255 | 3.9371 | 4.3480 |
| 60 | 5.3240 | 4.0628 | 4.5760 | 5.4731 | 5.3789 | 4.4536 | 4.6159 | 3.3690 | 4.1285 | 4.2616 |
| 61 | 5.6714 | 3.9456 | 4.9255 | 5.4799 | 5.9720 | 4.9862 | 4.5379 | 3.3960 | 3.8828 | 4.3770 |
| 62 | 5.5269 | 4.1654 | 5.0651 | 5.4775 | 5.6376 | 4.9704 | 4.5333 | 3.2539 | 3.7795 | 4.9461 |
| 63 | 5.0938 | 3.9820 | 5.0453 | 5.6892 | 5.9745 | 5.1790 | 4.5956 | 3.3637 | 3.7026 | 4.8199 |
| 64 | 5.3969 | 4.1939 | 5.1415 | 5.1002 | 5.6743 | 5.1407 | 4.5167 | 3.2129 | 4.3720 | 5.1858 |
| 65 | 5.5112 | 3.6890 | 4.8233 | 6.0842 | 5.7949 | 4.9186 | 4.4692 | 3.1404 | 4.0464 | 5.3110 |
| 66 | 5.4510 | 3.6692 | 5.0741 | 6.1851 | 5.3697 | 4.6970 | 4.5387 | 3.1985 | 4.3866 | 5.0291 |
| 67 | 5.4689 | 3.4472 | 4.6017 | 6.2876 | 5.9892 | 4.5865 | 4.2453 | 3.4404 | 4.1898 | 5.2317 |
| 68 | 5.4901 | 3.6215 | 4.9091 | 6.5108 | 6.1606 | 4.7976 | 4.3997 | 3.0931 | 4.3420 | 4.8265 |
| 69 | 5.9106 | 3.3059 | 4.7607 | 5.7631 | 6.2500 | 4.9039 | 4.4000 | 3.0234 | 4.2832 | 5.2216 |
| 70 | 6.3188 | 3.7484 | 4.7582 | 5.8317 | 6.2823 | 5.0227 | 4.5178 | 3.2114 | 4.1681 | 5.2895 |
| 71 | 6.4083 | 3.5065 | 4.9129 | 5.9132 | 5.9659 | 4.3988 | 4.4332 | 3.2653 | 4.5433 | 5.2181 |
| 72 | 5.5107 | 3.4430 | 4.9169 | 5.9339 | 6.3854 | 4.8940 | 4.6630 | 3.1644 | 4.2508 | 5.1940 |
| 73 | 5.7910 | 3.4023 | 4.9776 | 6.3303 | 5.7203 | 4.5005 | 4.5829 | 3.2471 | 4.7327 | 5.4321 |
| 74 | 5.8488 | 3.6069 | 5.7761 | 5.7873 | 6.1816 | 5.6385 | 4.4577 | 3.2119 | 4.1421 | 5.2013 |
| 75 | 5.6584 | 3.5592 | 5.8059 | 6.1551 | 6.3041 | 4.7237 | 4.5794 | 3.1168 | 4.0567 | 5.1100 |
| 76 | 6.6001 | 3.8965 | 5.9044 | 6.3955 | 6.3754 | 4.9181 | 4.6366 | 3.3660 | 4.1036 | 5.4655 |
| 77 | 5.8738 | 3.5958 | 5.5266 | 6.2198 | 7.0079 | 4.9212 | 4.6321 | 3.3757 | 3.9976 | 5.5541 |
| 78 | 5.6459 | 3.6433 | 6.4008 | 5.8277 | 6.4564 | 4.9484 | 4.7052 | 2.8646 | 3.8273 | 5.4325 |
| 79 | 5.3624 | 3.5307 | 5.7259 | 5.8203 | 6.1839 | 5.0430 | 4.4413 | 2.9507 | 3.7549 | 5.0749 |
| 80 | 5.8749 | 3.4775 | 6.9106 | 5.8769 | 6.3767 | 5.1753 | 4.4168 | 3.1178 | 4.1038 | 4.9373 |
| 81 | 5.9553 | 3.5619 | 7.0234 | 6.2072 | 6.6038 | 5.1023 | 4.2565 | 3.2677 | 4.0068 | 4.8389 |
| 82 | 5.7464 | 3.3933 | 6.5232 | 6.6199 | 5.8628 | 5.2075 | 4.2186 | 2.9652 | 3.7720 | 4.4652 |
| 83 | 5.7600 | 3.7525 | 5.8846 | 5.8822 | 5.4958 | 4.9616 | 4.5651 | 3.0157 | 4.2604 | 4.4755 |
| 84 | 5.8016 | 3.7955 | 5.9300 | 6.1331 | 5.5478 | 5.1933 | 4.3878 | 2.8786 | 4.3485 | 4.6099 |
| 85 | 5.7640 | 3.6540 | 6.4766 | 6.1908 | 5.8719 | 4.6482 | 4.6649 | 3.1113 | 4.1989 | 4.2987 |
| 86 | 5.8272 | 3.8147 | 5.5571 | 6.2038 | 5.8822 | 4.6630 | 4.4527 | 3.4357 | 4.3889 | 4.5175 |
| 87 | 5.7021 | 3.6406 | 5.7726 | 6.0652 | 5.9226 | 4.7116 | 4.3762 | 3.1538 | 4.2220 | 4.4257 |
| 88 | 5.5402 | 3.7826 | 5.6567 | 6.1505 | 6.3916 | 4.8748 | 4.2453 | 3.1670 | 4.0072 | 4.2471 |
| 89 | 5.4020 | 4.3907 | 5.5742 | 6.1118 | 6.2018 | 4.8703 | 4.1381 | 3.1927 | 4.1070 | 4.5134 |
| 90 | 5.8299 | 4.0970 | 6.3784 | 5.9934 | 6.1578 | 5.7759 | 4.2508 | 3.1232 | 4.0177 | 4.4835 |
| 91 | 5.4646 | 4.0397 | 5.8280 | 5.9988 | 5.7130 | 4.5421 | 4.0163 | 3.3186 | 4.4691 | 4.2875 |
| 92 | 5.3583 | 3.9973 | 5.6812 | 6.3398 | 6.4037 | 4.2295 | 3.9910 | 3.1269 | 4.4260 | 4.5816 |
| 93 | 5.2220 | 3.8318 | 5.8316 | 5.7527 | 5.8600 | 4.6433 | 4.2028 | 3.2496 | 4.4067 | 4.2393 |
| 94 | 5.8060 | 3.9039 | 5.6172 | 5.6106 | 5.9101 | 4.5421 | 4.2081 | 3.0246 | 4.3096 | 4.3864 |
| 95 | 5.5460 | 4.2605 | 5.6086 | 5.6096 | 6.3933 | 4.2439 | 4.2226 | 3.0547 | 4.6834 | 4.2603 |
| 96 | 5.6742 | 4.1332 | 6.5894 | 5.6028 | 5.5017 | 3.8676 | 4.1096 | 2.9626 | 4.1367 | 4.0430 |
| 97 | 5.7628 | 4.2952 | 6.1552 | 5.7404 | 4.8547 | 4.4562 | 4.0528 | 3.3108 | 3.9836 | 4.4113 |
| 98 | 5.5927 | 4.1213 | 6.7694 | 6.0943 | 5.1934 | 4.2331 | 4.0759 | 3.2331 | 4.3531 | 4.2589 |
| 99 | 5.7420 | 4.0826 | 5.3227 | 5.9883 | 5.9034 | 4.2226 | 3.7007 | 3.0598 | 4.5499 | 4.2191 |
| 100 | 5.7170 | 4.3356 | 5.0111 | 5.6667 | 5.9973 | 4.4747 | 3.8467 | 3.2181 | 4.5989 | 4.1444 |

**Table S2.** Human serum samples without neutralizing antibodies against SARS-CoV-2. The absence of neutralizing antibodies was confirmed using the cPass SARS-CoV-2 Neutralization Antibody Detection Kit for the Wuhan, Mu, BA.1, and BA.2 variants of the virus. Percentages of neutralization below 30% were observed, indicating the lack of neutralizing antibodies. Three technical replicates were performed for each serum sample.

| **SERUM** | **% NEUTRALIZATION** | | | | | | | | | | | |
| --- | --- | --- | --- | --- | --- | --- | --- | --- | --- | --- | --- | --- |
|  | **Wuhan** | | | **Mu** | | | **BA.1** | | | **BA.2** | | |
| **S1** | **3.34** | **2.16** | **1.62** | **0.92** | **1.91** | **12.37** | **4.87** | -0.43 | 4.03 | -0.79 | -1.21 | 2.00 |
| S2 | -1.31 | -3.89 | 0.51 | -1.53 | -5.10 | -1.93 | 4.90 | -1.23 | 5.23 | 2.00 | 2.52 | -2.61 |
| S3 | 6.74 | 3.44 | 2.09 | 1.48 | 1.29 | 1.14 | 3.15 | 0.16 | 4.39 | 6.23 | 3.59 | 4.08 |

**Table S3.** Promising Blocking Proteins (BPs) selected for further study. It includes the amino acid sequences, molecular weights (MW), and isoelectric points (IP) of the candidates, as determined by https://web.expasy.org/compute_pi/.

| **Name** | **Amino acid sequence** | **MW kDa** | **IP** |
| --- | --- | --- | --- |
| hACE2 | STIEEQAKTFLDKFNHEAEDLFYQSSLASWNYNTNITEENVQNMNNAGDKWSAFLKEQSTLAQMYPLQEIQNLTVKLQLQALQQNGSSVLSEDKSKRLNTILNTMSTIYSTGKVCNPDNPQECLLLEPGLNEIMANSLDYNERLWAWESWRSEVGKQLRPLYEEYVVLKNEMARANHYEDYGDYWRGDYEVNGVDGYDYSRGQLIEDVEHTFEEIKPLYEHLHAYVRAKLMNAYPSYISPIGCLPAHLLGDMWGRFWTNLYSLTVPFGQKPNIDVTDAMVDQAWDAQRIFKEAEKFFVSVGLPNMTQGFWENSMLTDPGNVQKAVCHPTAWDLGKGDFRILMCTKVTMDDFLTAHHEMGHIQYDMAYAAQPFLLRNGANEGFHEAVGEIMSLSAATPKHLKSIGLLSPDFQEDNETEINFLLKQALTIVGTLPFTYMLEKWRWMVFKGEIPKDQWMKKWWEMKREIVGVVEPVPHDETYCDPASLFHVSNDYSFIRYYTRTLYQFQFQEALCQAAKHEGPLHKCDISNSTEAGQKLFNMLRLGKSEPWTLALENVVGAKNMNVRPLLNYFEPLFTWLKDQNKNSFVGWSTDWSPYADQSIKVRISLKSALGDKAYEWNDNEMYLFRSSVAYAMRQYFLKVKNQMILFGEEDVRVANLKPRISFNFFVTAPKNVSDIIPRTEVEKAIRMSRSRINDAFRLNDNSLEFLGIQPTLGPPNQPPVSIWLIVFGVVMGVIVVGIVILIFTGIRDRKKPTPLLGKSWLTAILKD | 88.5399 | 5.44 |
| BP1 | KQSTIEEQAKTFLDKFNHEAEDLFYQSSLASANYNTNITEENVQNMNNAGDKWSAFLKEQSTLAQMYPLQEIQNLTVKLQLQALQQNSRQDQKAVCHPTAWDLGKGDFRILMCTKVTMDDFLTAHHEMGHIQYDMAY | 15.8247 | 5.07 |
| BP2 | IEEQAKTFLDKFNHEAEDLFYQSSLASWNYNTNITEENVQNMNNAGDKWSAFLKEQSTLAQMYPLQEIQNLTVKLQNGTIYSTGTQGFSENSMLTSKAVCHPTAWDLGKGDFRILMCT | 13.4259 | 4.72 |
| BP3 | KQSTIEEQAKTFLDKFNHEAEDLAYQSSLASANYNTNITEENVQNMNNAGDKWSAFLKEQSTLAQMYPLQEIQNLTVKLQLQALQQNSVAFKEAEKFIRQDQKAVCHPTAWDLGKGDFRILMCTKVTMDDFLTAHHEMGHIQYDMAY | 16.9119 | 5.13 |
| BP4 | TIEEQAKTFLDKFNHEAEDLFYQSSLASWNYNTNITEENVQNMNNAGDKWSAFLKEQSTLAQMYPLQEIQNLTVKLQLQALQQNSGSGAVCHPTAWDLGKGDFRILSCTKVTMDDFLTAHHEMGHIQYDMAY | 15.0977 | 4.76 |
| BP5 | STIEEQAKTFLDKFNHEAEDLFYQSSLASWNYNTNITEENVQNMNNAGDKWSAFLKEQSTLAQMYPLQEIQNLTVKLQLQALQQNSRQDQKAVCHPTAWDLGKGDFRILMCTKVTMDDFLTAHHEMGHIQYDMAY | 15.6835 | 4.92 |
| BP6 | IEEQAKTFLDKFNHEAEDLFYQSSLASWNYNTNITEENVQNMNNAGDKWSAFLKEQSTLAQMYPLQEIQNLTVKLQNGTIYSTGTQGFWENSMLTQKAVCHPTAWDLGKGDFRILMCTKVTMDD | 14.2559 | 4.63 |
| BP8 | IEEQAKTFLDKFNHEAEDLFYQSSLASWNYNTQITEENVQNMNNAGDKWSAFLKEQSTLAQMYPLQEIQQLTVKLQNGTIYSTGTQGFSENSMLTSKAVCHPTAWDLGKGDFRILMCT | 13.4539 | 4.72 |
| BP9 | TIEEQAKTFLDKFNHEAEDLFYQSSLASWNYNTQITEENVQNMNNAGDKWSAFLKEQSTLAQMYPLQEIQQLTVKLQLQALQQNSGSGAVCHPTAWDLGKGDFRILSCTKVTMDDFLTAHHEMGHIQYDMAY | 15.1258 | 4.76 |
| BP10 | IEEQAKTFLDKFNHEADELFYQSSLASWNYNTQITEENVQNMNNAGDKWSAFLKEQSTLAQMYPLQEIQQLTVKLQNGTIYSTGTQGFSENSMLTSKAVCHPTAWDLGKGDFRILMCT | 13.4539 | 4.72 |
| BP11 | TIEEQAKTFLDKFNHEADELFYQSSLASWNYNTQITEENVQNMNNAGDKWSAFLKEQSTLAQMYPLQEIQQLTVKLQLQALQQNSGSGAVCHPTAWDLGKGDFRILSCTKVTMDDFLTAHHEMGHIQYDMAY | 15.1258 | 4.76 |
