# Supplementary figures and images for: In Silico Design of miniACE2 Decoys with In Vitro Enhanced Neutralization Activity against SARS-CoV-2, Encompassing Omicron Subvariants
Source: Int J Mol Sci. 2024 Oct 8;25(19):10802. doi: 10.3390/ijms251910802 (PMC11476394; doi:10.3390/ijms251910802)

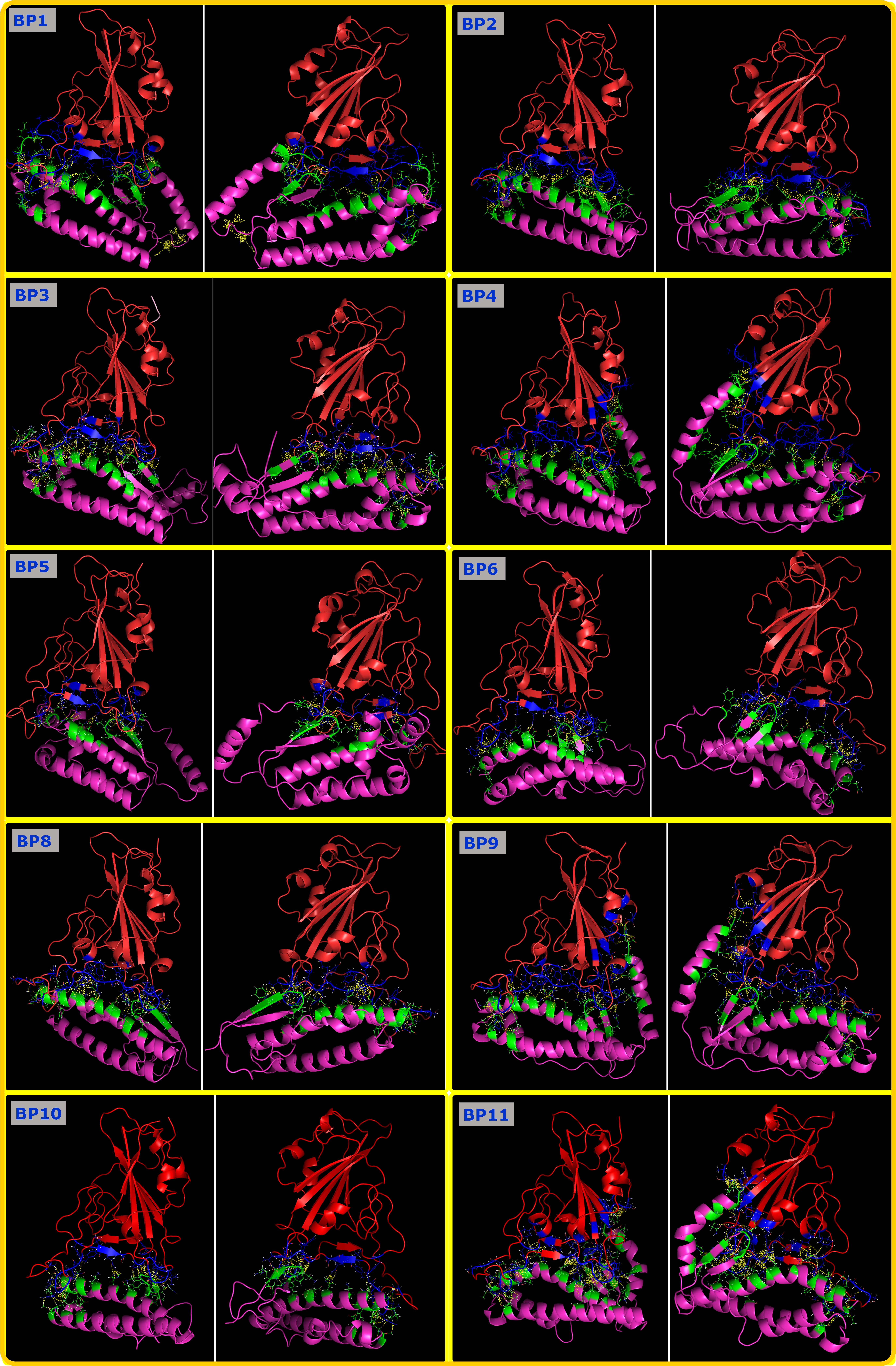

Supplement: Supplementary file 1 [file ijms-25-10802-s001.zip › Supplementary files/Supplementary figure 1..tif]

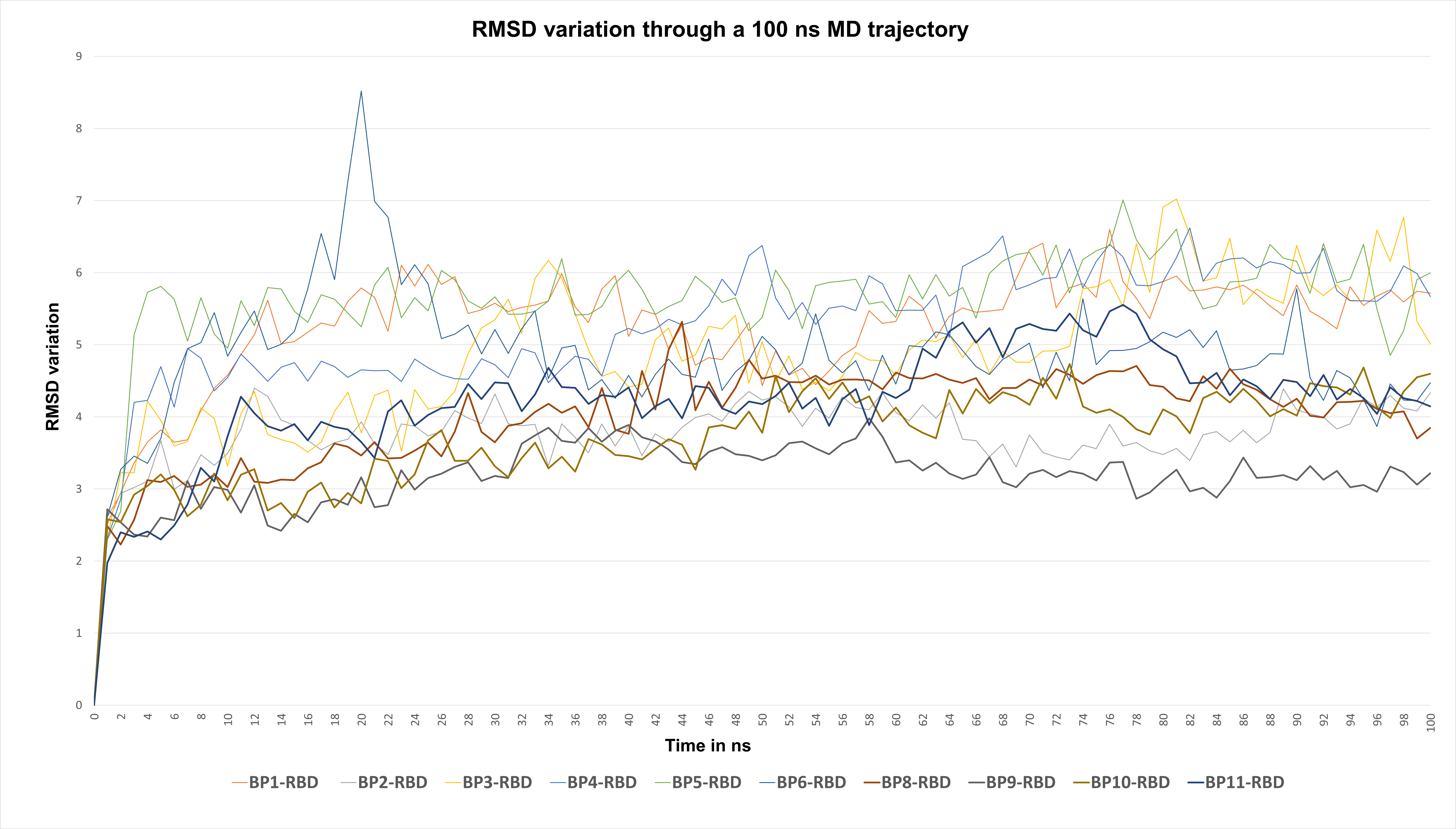

Supplement: Supplementary file 1 [file ijms-25-10802-s001.zip › Supplementary files/Supplementary figure 2.tif.png]

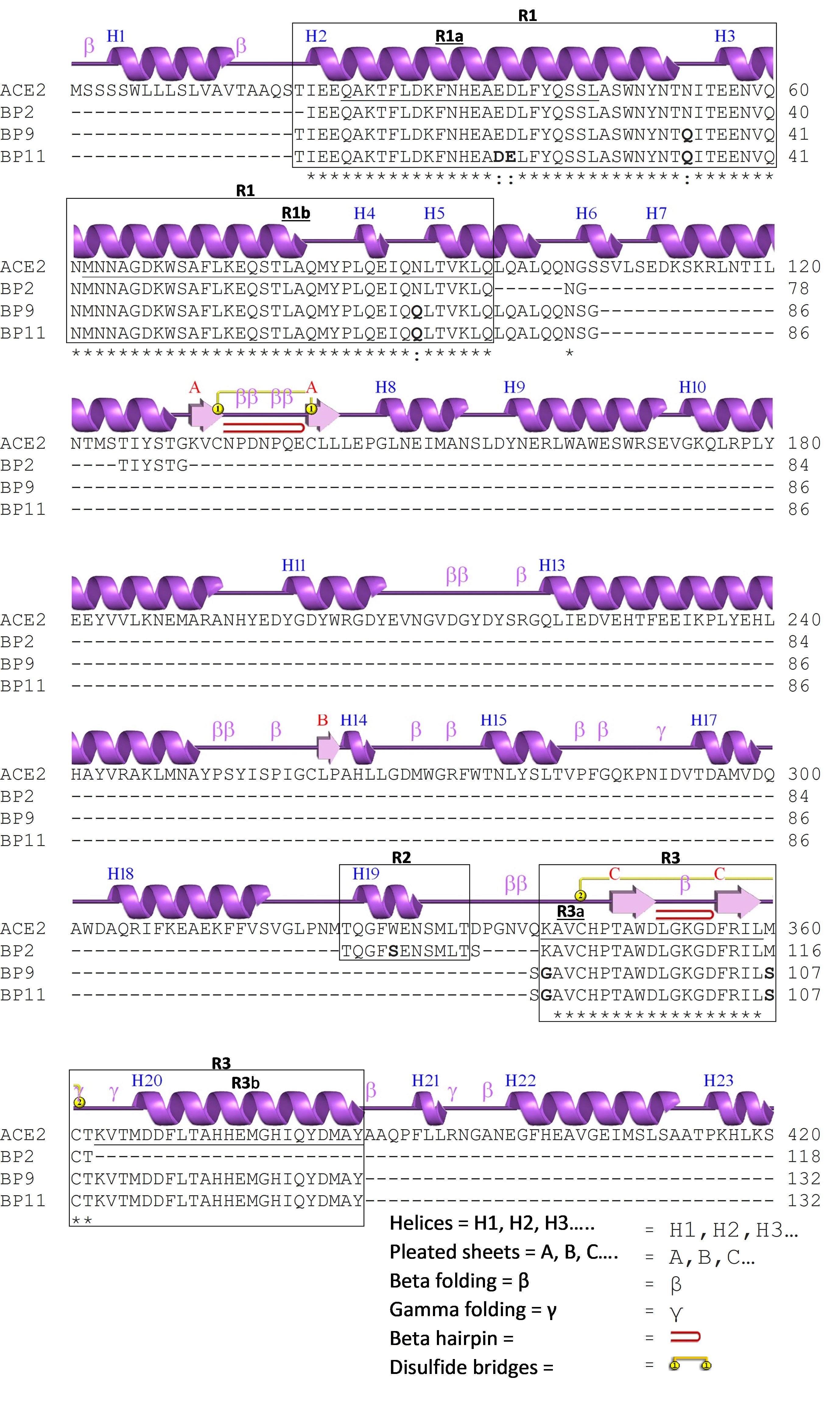

Supplement: Supplementary file 1 [file ijms-25-10802-s001.zip › Supplementary files/Supplementary figure 3..jpg]

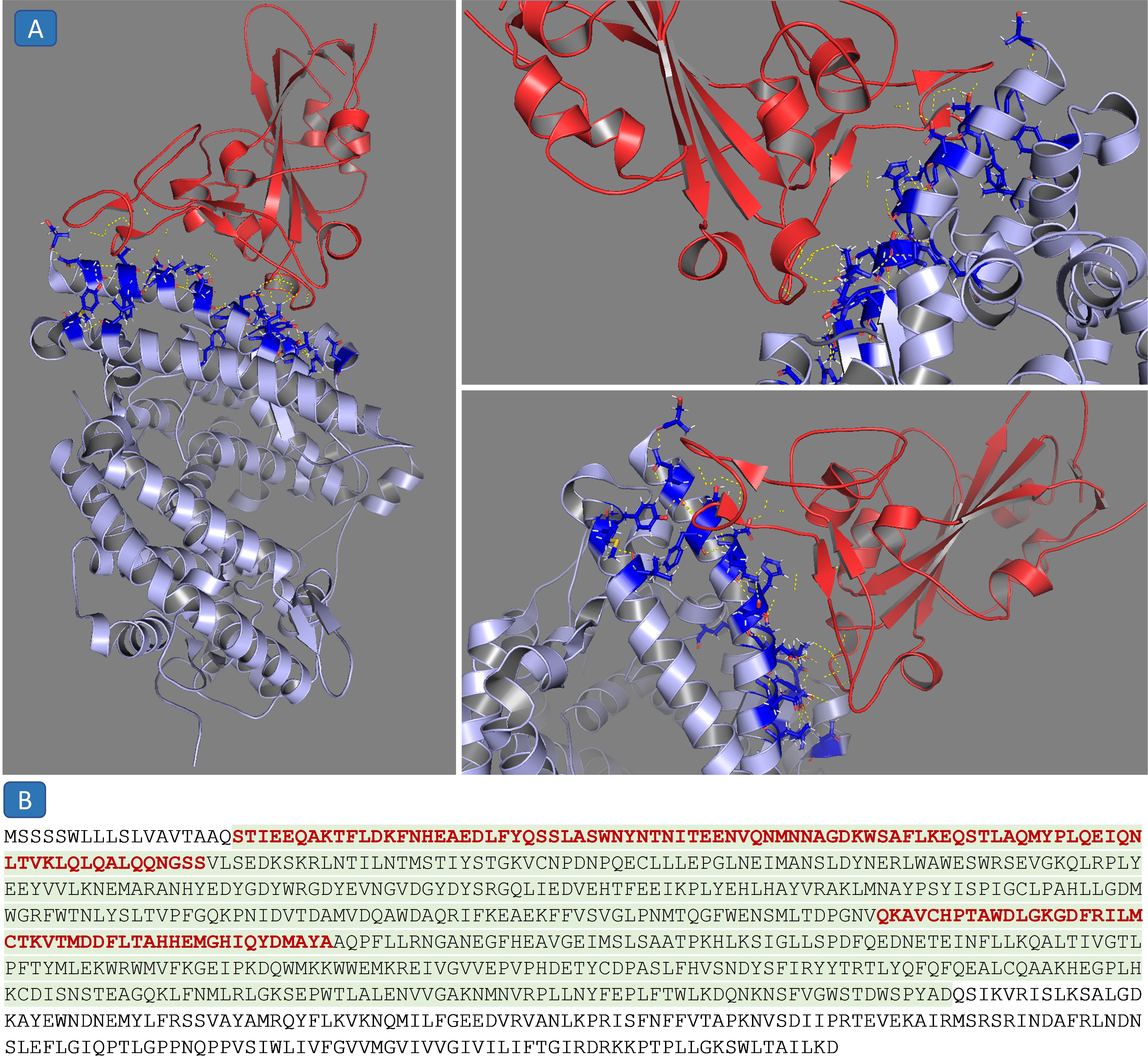

Supplement: Supplementary file 1 [file ijms-25-10802-s001.zip › Supplementary files/Supplementary figure 4..jpg]

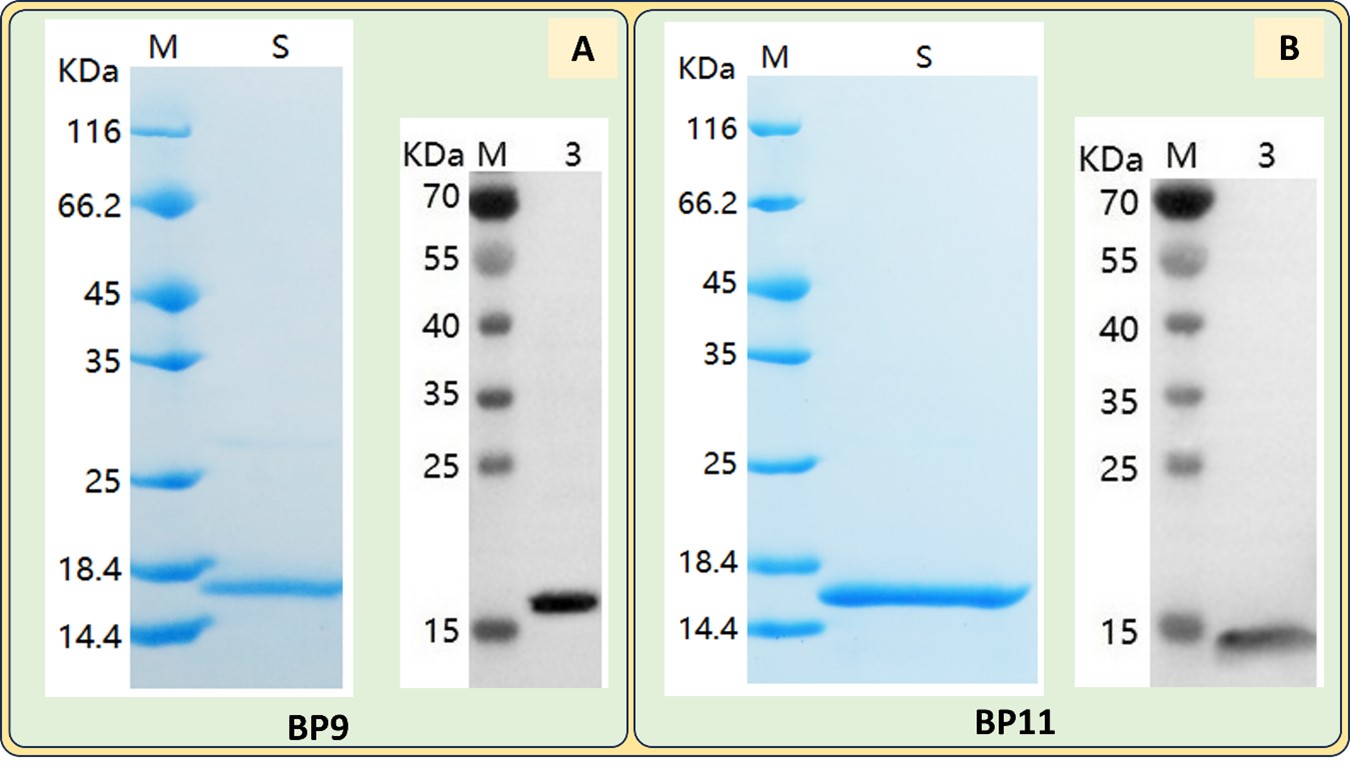

Supplement: Supplementary file 1 [file ijms-25-10802-s001.zip › Supplementary files/Supplementary figure 6..jpg]

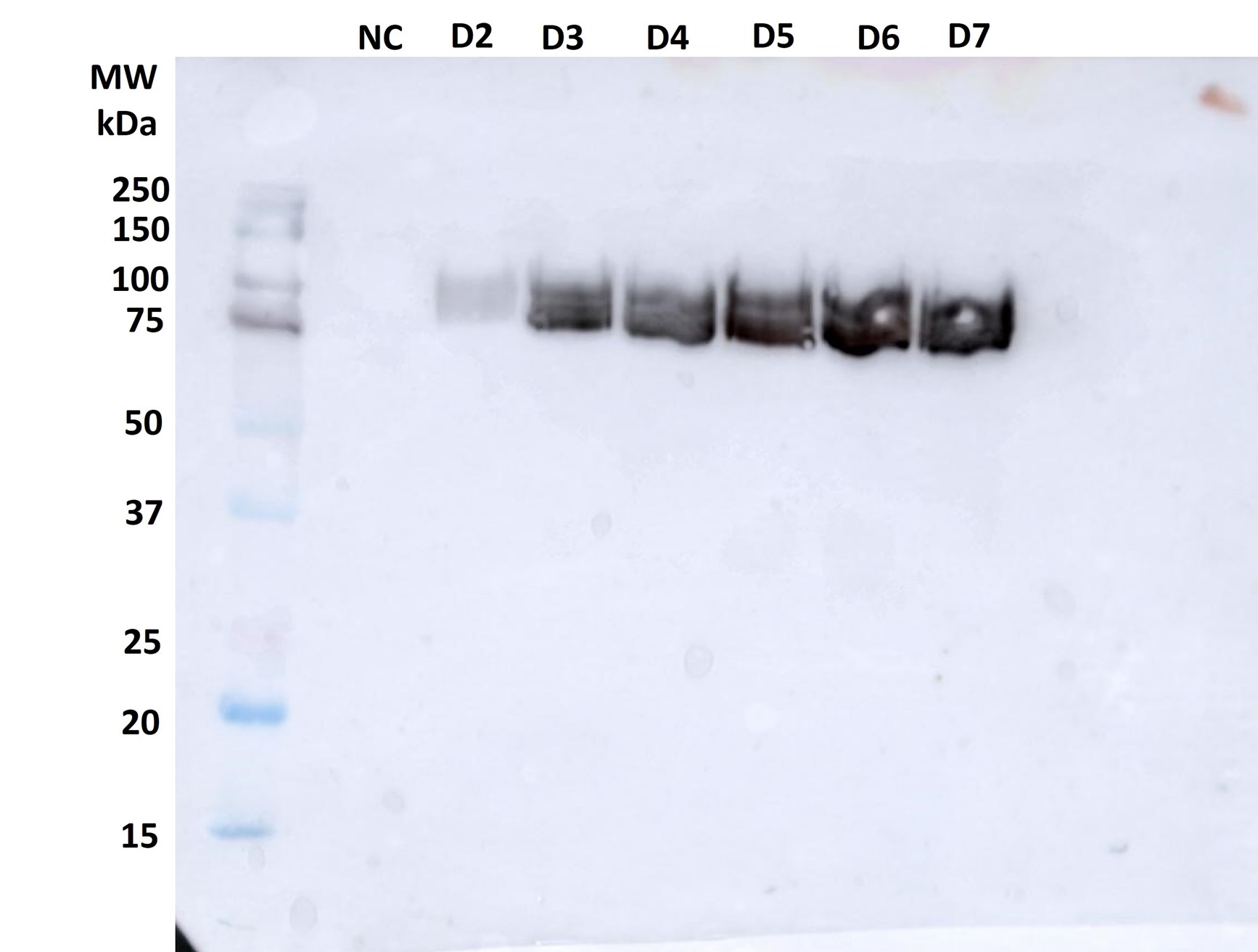

Supplement: Supplementary file 1 [file ijms-25-10802-s001.zip › Supplementary files/Supplementary figure 7..jpg]
